# Artificial Intelligence in Medical Writing: Addressing Untouched Threats

**DOI:** 10.31662/jmaj.2024-0268

**Published:** 2024-12-06

**Authors:** Shigeki Matsubara

**Affiliations:** 1 Department of Obstetrics and Gynecology, Jichi Medical University, Tochigi, Japan; 2 Department of Obstetrics and Gynecology, Koga Red Cross Hospital, Koga, Japan; 3 Medical Examination Center, Ibaraki Western Medical Center, Chikusei, Japan

**Keywords:** artificial intelligence, ChatGPT, human touch, paper, writing

## Abstract

The advantages and disadvantages of the use of generative artificial intelligence, such as ChatGPT, in medical writing have been widely discussed; however, two concerns remain largely unexplored. The first involves “human touch,” such as personal anecdotes and experiences. This touch often distinguishes human-written papers from those generated by ChatGPT as ChatGPT cannot independently access personal experiences. Although ChatGPT may mimic humanlike behavior, including the incorporation of a human touch, it lacks genuine emotions. With the lack of established guidelines on the acceptable levels of ChatGPT use and imperfect detection tools, many authors fear that their work could be perceived as overly reliant on ChatGPT. I worry that writers may artificially insert forced personal touches simply to assert their own writing. The second concern is the authors’ worry and doubt about whether to use ChatGPT and, if so, to what extent, which may disrupt their reflective and quiet writing process. While I acknowledge the lack of empirical data, I offer practical suggestions to balance the benefits of ChatGPT assistance and the preservation of the integrity of human writing in medical publications.

## First of All: Not a Luddite

First, I am not a Luddite. The Luddites were skilled workers in early 19th-century England who destroyed machines they felt were taking their jobs. They were frustrated about honing their skills for years only to be replaced by machines. While this historical event has various interpretations, my aim is not to oppose new technology but to discuss how we can effectively use it.

## What Has Been Hitherto Mainly Discussed regarding Artificial Intelligence in Medical Writing?

For simplicity, and considering its current wide usage, I refer to generative artificial intelligence (AI) as “ChatGPT.” Published papers on ChatGPT (OpenAI Inc., USA) in medical writing often address similar points ^(1)^: i) ChatGPT generates readable and reasonable manuscripts; ii) ChatGPT-generated manuscripts can contain inaccuracies, thereby requiring human oversight; iii) ChatGPT use must be fully disclosed; iv) journals should implement detectors for ChatGPT-generated manuscripts; and v) ChatGPT use raises concerns regarding plagiarism owing to its reliance on preexisting phrases. I want to highlight untouched issues: the potential of ChatGPT to alter authors’ mindsets.

## A “Human Touch” Characterizes a Human-written Paper, Right?

I previously reported this issue ^(2)^, and thus, I will briefly address it. I initially believed that “human touch” could differentiate a human-written from a ChatGPT-generated manuscript ^(2)^. Human touch involves personal anecdotes, experiences, and thoughts rooted in those experiences. I believe that this touch enhances the enjoyment of reading and writing, which is why I often include it in my papers ^(2)^. Although humans are the likely creators of this element, ChatGPT relies on accessible preexisting knowledge and thus cannot access personal anecdotes, making it less likely to produce a genuine human touch. A recent study reported that ChatGPT-generated manuscripts (essays for resident application) with this touch, depending on the input ^(3)^. However, I currently believe that skilled writers prefer to write these themselves rather than relying on ChatGPT. This is because ChatGPT may imitate humanlike behavior but does not possess, and thus may not generate, “genuine emotions.” At present, writers with less experience may not even think to add a human touch, when this issue has not yet been fully recognized. Thus, I currently believe that the thoughtful inclusion of human touch can still distinguish human-written from ChatGPT-generated manuscripts. This touch may serve as a “marker” of a human-written manuscript ^(2)^. What about the future?

## Untouched Concern about Paper Writing in the ChatGPT Era: Forcibly Adding a Human Touch

Various AI (ChatGPT) detection tools (AI-Detectors) have been introduced; however, data indicate that no AI-Detectors are perfect. For example, Flitcroft et al. ^(4)^ reported that completely AI-generated manuscripts had a higher average probability (43.5%) of being AI-generated, but this probability displayed a wide range (12.0%~99.9%) in a manuscript-by-manuscript manner. Notably, genuinely human-written manuscripts had an average probability of 9.4% of being AI-generated, exhibiting significant differences between the detectors. Only 0.4% of human-written manuscripts were detected as having 0% probability of being AI-generated. Notably, AI-Detectors significantly more often misidentified English nonnative-written manuscripts as ChatGPT-generated than native-written ones ^(5)^. This means that even if one writes independently without using ChatGPT, AI-Detectors may mistakenly judge human-written manuscripts as AI-generated ^(4), (5)^; this concern may be heightened for English nonnative authors’ writing ^(5)^. Taken together, this may lead authors, particularly nonnative ones, to want to avoid false accusations.

Everyone wants to claim, “I wrote this, not ChatGPT,” partly due to fear of such accusations (whether true or false) and because they understand that writing is a fundamental human skill ^(2)^. The use of ChatGPT might imply a lack of writing ability. Thus, once the “well-written” “human touch” becomes widely recognized as a marker of human-written manuscripts, authors may feel pressured to forcibly involve this touch just to prove “I wrote this.” This could resemble what happens in some entrance examination interviews where personal anecdotes are often expected, such as “My grandmother had cancer, so I decided to become a doctor.” In the ChatGPT era, I am concerned that medical publications might be filled with such forced personal touches (whether human-written or ChatGPT-generated), making them more annoying than enjoyable for readers.

## Another Untouched Concern: Worrying about Using or Not Using ChatGPT

I believe that many seasoned doctors have experienced the struggle of writing papers. The cycle of writing, submitting, and facing rejection serves as on-the-job training. These years of effort shaped them into independent writers, and then ChatGPT emerged. ChatGPT enables even beginners to produce readable papers.

Science thrives on originality. Some may argue that originality is essential for ideas only and not for writing style. However, when we express new ideas, our writing style naturally develops a personal flair. No one who has spent years refining their writing skills wants their work to sound like a beginner with ChatGPT. I believe that writing is a fundamental human endeavor. It nurtures our writing skills. Before ChatGPT, one must write fundamentally in one’s tone, whether they had no aid from anyone, received help from senior writers, or used commercial English writing services. At present, one must always be concerned about whether to ask ChatGPT’s aid or to write independently. ChatGPT consistently appeals to us and draws us in, always there beside us. There is always the temptation to “rely on ChatGPT; it will save time and still sound like your writing with appropriate input.” This temptation may be stronger for writers with less experience who have not yet developed their style. Irrespective of whether one is an experienced writer or less experienced in writing, day by day, paper by paper, the struggle to write independently may seem futile, and the temptation to heavily rely on ChatGPT grows.

Discussion continues to what extent ChatGPT use is allowed in paper writing ^(1)^, and I do not claim that genuinely human-written papers are inherently better than ChatGPT-generated or ChatGPT-aided ones. Considering the easy availability of ChatGPT, prohibiting ChatGPT use in paper writing is impractical. However, we can at least say the following: after ChatGPT, a concern arises: “to use or not to use ChatGPT and, if so, to what extent.” This can be annoying and may sometimes even disrupt the quiet writing process.

## Overcoming the Untouched Threats: My Proposal

Thus, the untouched concerns are i) the risk of inserting human touch merely to assert that a paper is human-written and that ii) one may be concerned about whether to use ChatGPT or not and, if so, to what extent. To address them, I propose a structured approach to using ChatGPT that preserves the integrity of the writer’s voice.

First, write papers as before the ChatGPT era. If one believes that adding a personal touch would enrich it, do so. Second, revise the manuscript numerous times, refining it until no further improvements can be made. Third, seek feedback from coauthors or colleagues and incorporate their suggestions. Fourth, use Grammarly (or some grammar-checking tools) to check for grammar and stylistic issues. Finally, once the manuscript is complete, use ChatGPT at this stage.

The effective use of ChatGPT requires a specific approach. If one simply asks it to “edit my manuscript,” you may end up with an extensively edited paper that does not feel like one’s own. Instead, input precise instructions, such as the following:

・“I want to retain my writing style.”

・“I don’t need sophisticated English.”

・“Only correct grave errors or peculiar expressions.”

Input the entire manuscript first so that ChatGPT can understand the context. Then, input it paragraph by paragraph for editing. This approach can help maintain one’s style. In addition, if one believes the original phrasing is better, ask ChatGPT, “Is my version incorrect?” It will often say, “Yours is also OK.” One can use one’s original phrases with confidence. I recommend avoiding words that require a dictionary check as one might not fully understand their meaning or the correctness of their usage in one’s context. Instead, ask ChatGPT to “tell me more plain words.”

I believe such usages of ChatGPT will help overcome two untouched concerns. “You” wrote it and thus one need not fear of being accused of “dependence on ChatGPT”: one need not forcibly involve human touch. One may have asked help from ChatGPT, but its use was just like Grammarly. One can say “this paper is my own.”

## Use ChatGPT as One of Your Helpers in Writing

Because authors have varying levels of writing experience and proficiency, a one-size-fits-all approach does not seem practical. My proposal is based on my four-decade paper writing practice, and to my knowledge, there is no objective data to support my proposal. I only wish to provide some hints for using ChatGPT in paper writing, and I do not intend to force anyone to employ this proposal.

No one can predict the future of academic publishing, but at least for the time being, I believe we should avoid using ChatGPT “from the outset of the writing process.” Heavily relying on ChatGPT may risk losing the joy of writing and the opportunity to develop as an independent writer.

## Article Information

### Conflicts of Interest

None

### Acknowledgement

A part of the present concept was previously described and was cited.

### Author Contributions

S. Matsubara: Manuscript writing.

### Approval by Institutional Review Board (IRB)

Not applicable

### Patient Anonymity

Not applicable.

### Informed Consent

Not applicable.

### Data Availability

Data sharing is not applicable to this article, as no new data were created or analyzed in this study.
